# Knowledge and Opinions of French Dental Students in Operative Dentistry – Management of Deep Carious Lesions

**DOI:** 10.3290/j.ohpd.b2403521

**Published:** 2021-12-08

**Authors:** Marie-Agnès Gasqui, Laurent Laforest, Justine Le Clerc, Romain Ceinos, Florence Chemla, Valérie Chevalier, Pierre Colon, Florence Fioretti, Alexis Gevrey, Olivia Kérourédan, Delphine Maret, Caroline Mocquot, Canan Ozcan, Fabienne Perez, Elodie Terrer, Yann-Loïg Turpin, Reza Arbab-Chirani, Sophie Doméjean, Dominique Seux

**Affiliations:** 1 Lecturer, Multimaterials and Interfaces Laboratory (UMR 5615), Faculty of Dentistry, University of Lyon 1, Lyon, France. Made a synthesis of the results and wrote the manuscript; read and approved the manuscript.; 2 Lecturer, Faculty of Dentistry, University of Lyon 1, Lyon, France. Made a synthesis of the results and wrote the manuscript. Read and approved the manuscript.; 3 Lecturer, Multimaterials and Interfaces Laboratory (UMR 6226), Faculty of Dentistry, University of Rennes 1, Rennes, France. Read and approved the manuscript.; 4 Lecturer, Faculty of Dentistry, Côte dʼAzur University, Nice, France, Nice University Hospital, Odontology Center, Nice, France; UMR 7268, Bio-cultural anthropology, Ethics and Health Law (ADES), Aix-Marseille University, Marseille, France. Read and approved the manuscript.; 5 Professor, Faculty of Dentistry, University Paris Descartes; Department of Dentistry, Charles Foix Hospital, APHP Paris, France. Read and approved the manuscript.; 6 Lecturer, University of Bretagne Occidentale, Brest, France; UMR CNRS 6027, IRDL, F-29200 Brest, France; Dental University Hospital of Brest, France. Read and approved the manuscript.; 7 Professor, Paris University, AP-HP Rothschild hospital Paris, LMI, UMR CNRS 5615 Lyon 1, Lyon, France. Read and approved the manuscript.; 8 Lecturer, Faculty of Dentistry, Strasbourg University, Department of Medicine and Dental Surgery, Strasbourg University Hospital, UMR INSERM 1260, Strasbourg, France. Read and approved the manuscript.; 9 Lecturer, Faculty of Dentistry, University of Nancy, France. Read and approved the manuscript.; 10 Lecturer, Faculty of Dentistry, University of Bordeaux, Bordeaux, France; University Hospital of Bordeaux, Department of Dental Surgery, Bordeaux, France; INSERM, Bioingénierie Tissulaire, U1026, Bordeaux, France. Read and approved the manuscript.; 11 Lecturer, Faculty of Dentistry, University Hospital of Toulouse, Laboratoire AMIS, UMR 5288 CNRS, Toulouse, France. Read and approved the manuscript.; 12 Lecturer, Department of Conservative Dentistry, Faculty of Dentistry, University of Reims Champagne-Ardennes, France. Read and approved the manuscript.; 13 Professor, INSERM, UMR 1229, RMeS, Regenerative Medicine and Skeleton, ONIRIS, University of Nantes, Faculty of Dentistry, University Hospital, PHU4 OTONN, Nantes, France. Read and approved the manuscript.; 14 Lecturer, Faculty of Dentistry, Aix-Marseille University, IRD, MEPHI, IHU Méditerranée Infection, Marseille, France. Read and approved the manuscript.; 15 Lecturer, University of Rennes, University Hospital of Rennes, Department of Conservative Dentistry, France. Read and approved the manuscript.; 16 Professor, Faculty of Dentistry, University of Brest, LaTIM UMR 1101 INSERM, 29238, Brest, France. Read and approved the manuscript.; 17 Professor, Faculty of Dentistry, Clermont Auvergne, Center in Clinical Odontology EA 4847, Clermont-Ferrand, University Hospital Estaing, Clermont-Ferrand, Service d’Odontologie, Clermont-Ferrand, France. critically reviewed the manuscript and made suggestions; read and approved the manuscript.; 18 Professor, Faculty of Dentistry, Department of Conservative Dentistry, University of Lyon 1, Lyon, France. Made a synthesis of the results and wrote the manuscript; read and approved the manuscript.

**Keywords:** deep carious lesions, dental education, dental students, minimal intervention

## Abstract

**Purpose:**

A questionnaire survey was recently undertaken among French dental students (FDSs) to investigate their practices, knowledge and opinions in various domains of minimal intervention (MI) in cariology. The present work focuses on management of deep carious lesions (DCLs).

**Materials and Methods:**

The questionnaire was administered (Spring 2018) to all the fifth-year students of the 16 French dental schools. Descriptive analyses were performed.

**Results:**

Among 1370 FDSs (response rate: 84.5%), hardness was the most commonly reported criterion for assessing the endpoint of carious tissue removal (53.9%), followed by firm dentin (40.0%). Regarding FDSs’ opinion of leaving carious dentine under a restoration, 41.9% of the respondents agreed that carious tissues should always be removed completely. For an asymptomatic tooth with DCLs and exposed pulp, direct pulp capping was mainly chosen (93.9%). In a clinical case correctly diagnosed as a reversible pulpitis by 79.7% of respondents, nearly half of FDSs chose a one-step complete excavation (48.3%) followed by selective excavation (25.1%), then two-step complete excavation (20.9%) and a minority (5.7%) opted for pulpal therapy (biopulpotomy or endodontic treatment).

**Conclusion:**

The present results suggest an inadequate dissemination of MI concepts among FDSs towards DCL management. The present results show the need for a harmonisation and a reinforcement of teaching evidence-based MI according to the latest European recommendations.

The contemporary approach to caries management (minimal intervention or MI) has dramatically changed and new concepts in cariology have increasingly gained interest in dental practice. Currently, only cavitated carious lesions which are either non-cleansable or can no longer be sealed need restorative interventions.^18^ Thus, MI aims to control existing carious lesions and to preserve hard dental tissues whenever possible. MI enables the preservation of the tooth, avoiding invasive therapeutic options, including an expensive re-restoration circle.^14,18^

The traditional management of deep carious lesions (DCLs) relies on the non-selective removal of all soft dentine until hard dentine is felt. Nonetheless, this approach can be challenging given the risk of pulp exposure and the occurrence of subsequent complications, namely hypersensitivity, pulp exposure and endodontic procedures.^19^ The International Caries Consensus Collaboration currently advocates for alternative approaches to DCLs, avoiding pulp exposure and based on selective removal of carious dentine or stepwise removal in adults. Conversely, complete caries removal is henceforth considered as over-treatment.^10,14,18^

Recent studies show that therapeutic approaches based on MI have only partially entered dental clinical practice, more particularly when managing DCLs.^4,12,19,24^

 In addition to the insufficient knowledge of some clinicians,^8^ different barriers related to health systems persist to allow a better implementation of MI in dental care.^6^

University training is a key period to acquire therapeutic concepts. In view of optimising the dissemination of MI in dental care and of tailoring university training on MI in cariology, there is a need to document dental students’ (FDSs) beliefs and therapeutic behaviours with respect to the DCL management. We expected that current French dental students (FDSs) would be satisfactorily sensitised to MI precepts and inclined to use them.

A study, the first of its kind in France, was thus undertaken to investigate the knowledge and opinions of FDSs at a national level about several areas of MI in cariology, namely caries risk assessment, dental sealants (preventive and therapeutic), restorative thresholds and strategies for proximal and occlusal lesions, and DCL management. The present manuscript focuses on DCL management.

## Materials and Methods

The methodology of the study has already been described.^13^ Briefly, a questionnaire survey was conducted in May–June 2018 among the fifth-year FDSs (penultimate year before graduation; n = 1,370) of the 16 public French dental schools (Bordeaux, Brest, Clermont-Ferrand, Lille, Lyon, Marseille, Montpellier, Nancy, Nantes, Nice, Paris Descartes, Paris Diderot, Reims, Rennes, Strasbourg and Toulouse). As the present study only focused on FDSs’ learning outcomes, no approval of ethical committees was required according to French regulation.

All FDSs were included, and there were no criteria of exclusion.

The questions administered to FDSs come from a questionnaire previously used for surveys among general dental practitioners (FGDPs).^4,7,8,11,12,19,21,22^ This questionnaire was anonymously self-administered to the FDSs in a specific session organised in each dental school. The present article is focused on the management of DCL.

First, respondents were invited to complete questions regarding routine approaches to treat DCLs, along with the reasons for their treatment preferences and knowledge-related attitudinal items. These questions were then followed by three clinical cases (CC) consisting of young patients with low carious risk, no medical history of allergies or use of medications. Patients’ general and dental history, and oral hygiene practices were documented. These three patients complained about the occurrence of pain induced by chewing and/or by a cold stimulation in posterior teeth. In each CC, a clinical occlusal view before and after opening the lesion were provided, as well as a periapical radiograph. All carious lesions reached 75% of dentinal thickness, thus the risk of pulp exposure was a reality for the three cases. Further, respondents were invited to choose the most likely diagnosis with the most relevant treatment. The following subgroups were used for statistical analysis related to the CC treatment strategies: complete excavation (one or two steps) versus selective excavation versus pulp therapy (pulpotomy and endodontic treatment).

The data were entered into an Excel spread sheet by four persons (three dentists: DS, MAG, SD, and a Master’s student: LDB). The descriptive and statistical analyses were performed with SPSS (IBM SPSS Statistics Version 19).

## Results

### Participants’ Characteristics

All of the 16 French dental schools participated in the survey. Among 1,370 FDSs, a total of 1,158 fulfilled questionnaires (response rate: 84.5%) were collected. The number of FDSs per dental school ranged from to 26 to 122. The average age of the participants, at the time of the study, was 24.5 (±2.12) years-old (46.5% of men).

### Knowledge and Attitude on the Management of DCL

#### Criteria, methods and strategies for carious tissue removal

Table 1 presents the criteria and methods used by FDSs for caries tissue removal in DCLs. The most frequent responses for hardness criteria were hard dentine and firm dentine. Normal to yellow was the most cited item regarding colour. ‘Moisture’ was not a relevant criterion for most respondents.

**Table 1:** Criteria and methods used for carious tissue removal in deep lesions

Category	Criterion/method	Percentage of respondents
Criteria you routinely rely on to confirm satisfactory removal of caries in DCL		
Hardness (n = 1,138)		
	Soft	4.4% (n = 50)
	Leathery (firm)	40.0% (n = 456)
	Hard	53.9% (n = 613)
	Not relevant	1.7% (n = 19)
Colour (n = 1,141)		
	Heavily stained	10.7% (n = 122)
	Normal to yellow	53.5% (n = 610)
	Not relevant	35.8% (n = 409)
Moisture (n = 1,132)		
	Wet	1.6% (n = 18)
	Moist	12.6% (n = 143)
	Dry	29.1% (n = 642)
	Not relevant	56.7% (n = 642)
Excavation method		
n = 935	Diamond bur	24.1% (n = 225)
n = 1,062	Metal bur	85.2% (n = 905)
n = 917	Ceramic bur	28.7% (n = 263)
n = 864	Hand excavator	88.7% (n = 959)
n = 863	Chemo-mechanical	7.5% (n = 65)
n = 1,135	Rubber dam	96.2% (n = 1,092)
n = 1,116	Cavity disinfection	81.7% (n = 912)
n = 1,052	Dye solution	10.8% (n = 114)

Hand excavator and metal burs were the primary excavation methods by FDSs. Almost all students reported using a rubber dam and over 80% performed cavity disinfection.

#### Reasoning and decision-making

When asked for the reasons underlying their excavation strategy, about 80% of respondents knew that a certain number of microorganisms could be left because restorations seal carious tissue (Table 2). Similarly, 77.3% of students believed that pulpo-proximal carious dentine might be left to avoid pulp exposure. Conversely, some FDSs believed that cariogenic microorganisms needed to be removed completely, since residual caries might constitute a risk for the vitality of the pulp (41.9%) or for carious lesion progression (39.2%).

**Table 2:** FDSs’ attitude towards leaving carious dentine under a restoration^(a)^

	Disagreement	Neutral	Agreement
Cariogenic microorganisms need to be completely removed, as caries might progress otherwise (n = 1,142)	58.2% (n = 665)	2.5% (n = 29)	39.2% (n = 448)
Caries must always be completely removed, as residual caries is a risk for the vitality of the pulp (n = 1,142)	54.9% (n = 627)	3.2% (n = 37)	41.9% (n = 478)
A certain number of cariogenic microorganisms can be left behind, as intact restorations can seal and thus stop caries (n = 1,144)	16.0% (n = 183)	4.1% (n = 47)	79.9% (n = 914)
Caries near the pulp should be left to avoid pulp exposure (n = 1,143)	17.9% (n = 205)	4.7% (n = 54)	77.3% (n = 884)

^(a)^ Not all participants answered the question.

Table 3 represents the reasons guiding therapeutic options. Decisions were largely driven by recommendations from teachers or peers, and the good results obtained with the chosen therapy. Over half of the respondents reported recommendations by clinical studies or guidelines and ease of use and familiarity with the technique. Treatment decision was mainly oriented by the patient’s oral health, the volume of coronal dental substance loss, the patient’s general health and his/her age.

**Table 3:** FDSs’ reasons guiding different therapeutic options and treatment decisions

Category	Reason	Percentage of respondents
Main reasons guiding therapeutic options	Recommended by peers	73.7% (n = 851)
	Good results	70.6% (n = 816)
	Recommended by clinical studies	59.1% (n = 683)
	Recommended by clinical guidelines	52.4% (n = 605)
	Technical skills, easy to achieve	54.2% (n = 626)
	Recommended in books	33% (n = 381)
	Not an expensive technique	8% (n = 92)
Main factors influencing treatment decision	Patient’s oral health (particularly carious risk)	85.5% (n = 988)
	Importance of coronal loss	78.2% (n = 904)
	Patient’s general health	62.5% (n = 722)
	Patient’s age	59.3% (n = 686)
	Patient’s attitude and concern	55.1% (n = 637)
	Type of tooth	52.5% (n = 607)
	Tooth already restored	49.8% (n = 576)
	Amount of time necessary for the global treatment	16.5% (n = 191)

#### Asymptomatic vital tooth with a DCL

In a scenario of an asymptomatic tooth with a DCL and pulp exposure during carious tissue removal, 93.9% of respondents chose a direct pulp capping. In the same scenario (DCL and pulp exposure during carious tissue removal) with a symptomatic pulp, the most frequent responses were partial pulpotomy (53.9%) followed by endodontic treatment (38.8%).

Consistent with this vision of pulpal preservation, about 40% (38.4%) of respondents would perform other less invasive treatments rather than endodontic treatments on a symptomatic tooth with DCL, while 59.8% would perform endodontic treatment. In case of asymptomatic teeth with DCL, only a minority reported endodontic treatment as the first option (1.9%).

### Clinical Cases: Pulpal Diagnoses and Treatment Options

In CC 1, reversible pulpitis was the most reported diagnosis. Nearly half of FDSs chose a one-step complete excavation at the expense of other therapeutic options, notably partial excavation (Table 4). In CC 2, dentinal carious lesion was most frequently diagnosed by FDSs (60.9%), while almost all the others (38.6%) agreed with a reversible pulpitis in CC 2. In the same way, most FDSs selected a one-step complete excavation as the best option, while alternatives were marginally cited. In CC 3, two-thirds diagnosed a reversible pulpitis. FDSs who cited the one-step total excavation or pulpal therapy accounted for 65.4%, while partial or stepwise excavation were less mentioned: 19.6% and 15.0%, respectively. When restricting the analyses to FDSs who rightly diagnosed a reversible pulpitis, these trends were not affected (62.9% for one-step total excavation or pulpal therapy, 19.6% and 17.6% for partial and stepwise excavation, respectively).

**Table 4:** FDSs’ reported diagnoses and treatments for three clinical cases (CC)

Diagnosis and therapeutic for three CCs				
		CC 1	CC 2	CC 3
Diagnosis (a)	Dentinal carious lesion	18.6% (n = 210)	60.9% (n = 688)	21.9% (n = 245)
	Reversible pulpitis	79.7% (n = 897)	38.6% (n = 436)	66.0% (n = 739)
	Irreversible pulpitis	1.7% (n = 19)	0.5% (n = 6)	12.1% (n = 135)
Treatment (a)	Complete dentinal excavation in one step	48.3% (n = 544)	72.0% (n = 816)	24.5% (n = 277)
	Complete dentinal excavation in two steps	20.9% (n = 235)	9.9% (n = 112)	15% (n = 170)
	Partial dentinal excavation	25.1% (n = 282)	15.5% (n = 176)	19.6% (n = 221)
	Pulpotomy	3.7% (n = 42)	2.1% (n = 24)	23.6% (n = 267)
	Endodontic treatment	2.0% (n = 22)	0.5% (n = 6)	17.3% (n = 195)

^(a)^ A unique answer was expected.

## Discussion

The present study is the first of its kind and aims at providing an overview of the knowledge and opinions of fifth-year FDSs regarding DCL management. All the 16 French dental universities participated in the present study and a global satisfactory response rate was obtained (84.5%), making the results highly representative of the overall population of fifth-year French FDSs.

MI precepts related to DCL management appeared to be partially integrated by FDSs, both in terms of beliefs and declared behaviours. Satisfactorily, nearly four out of five FDSs agreed with leaving a certain amount of carious lesion near the dental pulp (77.3%) and with the absence of risk of carious lesion development when properly sealed (79.9%). Conversely, 41.9% of FDSs believed that all caries tissues needed to be removed (Table 2). The inconsistency of responses between these contradictory statements could reveal a misunderstanding in the formulation of the questions, or more worryingly in the concepts of MI. Besides ignorance or misunderstandings, FDSs’ non-adherence or mistrust toward the new MI recommendations could account for the common belief that leaving carious tissue is harmful. Former well-rooted beliefs could be hard to shed. The widespread habit of cavity disinfection reported in the study by FDSs (81.7%) reflects the lingering perception of carious lesion as an infection process, as also observed among many French and German GDPs (74% for both countries).^19^

Reversible pulpitis was the expected diagnosis for the three CC. Based on radiographic dentine involvement, expected treatment was selective removal to firm dentine in CC1 and CC2, since carious lesions were limited to the inner third of dentine. Conversely, partial excavation with selective removal to soft dentine was required in CC3, as carious lesions expanded over the inner-third of the dentine.^20^

Almost all FDSs diagnosed either a dentinal carious lesion or a reversible pulpitis for CC 1 and CC2 (Table 4). While MI approaches would have been recommended in these CCs,^10^ more particularly the selective removal to firm dentine,^1,18^ many FDSs would indicate a one-step complete dentinal excavation, 48.3 % for CC1 and 72% for CC2, respectively. Unfortunately, responses observed for partial dentinal excavation or two-step complete dentinal excavation were respectively provided by less than half of FDSs in both CCs (Table 4). Two-thirds of FDSs diagnosed a reversible pulpitis for CC3 while 12.1% wrongly opted for irreversible pulpitis. As in previous CCs, an evidence-based treatment would have been a selective removal to soft dentine or a stepwise approach given the absence of lingering pain.^18^ Regrettably, 65.4% of FDSs still preferred a one-step complete excavation or pulpal therapy. The unchanged conclusions after restricting the analyses to DSs who rightly diagnosed a reversible pulpitis (62.9%) suggests that a potential difficulty of differential diagnosis between reversible and irreversible pulpitis cannot affect our conclusions. Overall, these results suggest an inadequate assimilation of MI therapeutic options among FDSs for the treatment of DCL or a reluctance to use them. As it was the case for partial dentinal excavation, two-step complete excavation was relatively little selected in the different CCs (Table 4), although these therapeutic approaches are reliable treatments for well-defined DCL located in the pulpal quarter of the dentine.^10^

A majority of FDSs (53.9%) stated hardness of the cavity floor as a criterion for the endpoint of excavation (40.0% for firm dentine). This could reflect a reluctance of some FDSs to leave some remineralisable carious tissue near the pulp.^18^ Further, noticeable proportions of FDSs relied on pulp colour and moisture (64.2% and 43.3%, respectively), while these latter criteria are not considered as reliable by the International Caries Conference Consensus.^18^

Encouragingly, more than half of FDSs reported that their treatment decisions were driven by evidence-based clinical guidelines and studies (Table 3). However, improvements are required. The predominant influence of peer recommendations in the FDSs decision-making process (73.7%) merits interest as it suggests that a word-of-mouth effect could promote the dissemination of MI precepts among them. Patient’s oral health was a frequently cited clinical criterion, as it was the case for dentists in most countries.^12,21^ Another positive finding was the predominant proportion of rubber dam users, suggesting an effective dissemination of this recommendation by curricula (Table 1). Likewise, in case of pulp exposure during carious tissue removal on an asymptomatic tooth, direct pulp capping was the most commonly chosen option (93.9%). A similar, although slightly lower frequency of use, was observed among FGDPs (85%).^21^

A very frequent handling of excavators by FDSs emerged, a similar proportion was observed among Spanish GDPs.^5^

However, some methodological limitations should not be overlooked. The present study was conducted among the fifth-year FDSs while the sixth-year FDSs would have been a more intuitive target for the study population. This choice was motivated by the presence of an internship (similar to vocational training) during the sixth year (final year) of the dental course in France, which would have resulted in a noticeable loss of FDSs upon inclusion of the study. Although we believe that the questionnaire captured the perceived management of DCLs by students about to graduate and join the professional world of dentistry, differences in response patterns between fifth and sixth-year FDSs cannot be formally excluded. FDSs’ answers could be mainly influenced by their training and possibly by their current practice. Further, good knowledge of MI and adherence to corresponding behaviours by FDSs may not guarantee effective practice once they are in their future professional context. Indeed, the lack of current reimbursement of MI in the French health system tends to discourage preventive therapeutic behaviors^9^ and FGDPs may prioritise financial criteria. Only a global analysis was conducted because the objective of the study was not to make any comparisons between the different French universities. FDSs who opted for a more aggressive therapy were more inclined to believe in the detrimental effect of leaving soft dentine under a filling. MI therapeutic options related to pulpal diagnosis might not always be successful in the long-term because restorative treatment must be nuanced according to the loss of substance. Hence, monitoring the long-term success of MI treatments should be desirable in such cases.

The present data have implications. FDSs are the practitioners of the future and their appropriation of MI concepts for caries treatment is critical to promote any subsequent change in daily dental practice. Improving FDSs’ knowledge and behaviours with respect to MI requires a reinforcement of their training on this topic, in order to better adhere to recent courses in cariology^2,3,17^ and to the consensus for cavitated caries lesions.^18^ Further, harmonisation of training on MI is desirable between the different French universities for greater efficiency.^15,23^ In addition, French dental curricula should devote more time to the reading of scientific articles and recent evidence-based documents. A study would also be helpful to assess potential changes once improvements in FDSs training have been made.^16^

## Conclusion

In conclusion, this study highlighted the need to improve the sensitisation of FDSs about MI in the curricula, particularly for DCL management. Nevertheless, some changes are also needed in the French healthcare system. Health authorities should support MI to promote its use, especially since this could lead to a drop in the cost of care. National guidelines regarding MI are still missing and could encourage wider use of MI in dental practice. A reform of dental care is underway in France towards better reimbursement of restorative therapeutic approaches. This trend could allow us to be optimistic about a future integration of MI in the health insurance coverage of dental care in France.
